# Interband Consistency-Driven Structural Subspace Clustering for Unsupervised Hyperspectral Band Selection

**DOI:** 10.3390/s25237265

**Published:** 2025-11-28

**Authors:** Zengke Wang, Wenhong Wang

**Affiliations:** College of Computer Science, Liaocheng University, Liaocheng 252059, China; 2120170215@stu.lcu.edu.cn

**Keywords:** band selection, consistent representation, hyperspectral image classification, structural subspace clustering

## Abstract

In the classification applications of hyperspectral remote sensing images (HSIs), band selection is crucial for mitigating the curse of dimensionality while preserving the intrinsic physical information within HSIs. Although clustering-based band selection methods are widely applied, they often overlook the inherent physical properties of hyperspectral images. Such approaches typically operate in raw high-dimensional space, which is susceptible to noise and redundancy. This results in generated band combinations that fail to adequately characterize the spectral features of the underlying materials, leading to suboptimal band-grouping schemes. To address this, we propose a novel Interband Consistency-Constrained Structural Subspace Clustering (ICC-SSC) method. The core assumption is that the spectral characteristics of land cover inherently reside within a low-dimensional subspace, where bands within this subspace should exhibit strong physical consistency, which means that the spectral signatures of land covers show significant similarity across these bands. Driven by this physical interpretation, our method innovates in two ways. Specifically, we employ the $l_{1,2}$ norm in the self-representation model to discover the inherent grouping structure of the bands. This enforces a small set of common, representative basis bands to reconstruct others, effectively identifying the most physically informative bands that anchor these material-specific subspaces. In addition, we incorporate a total variance (TV) regularization term into the proposed model to capture the smoothing characteristics between adjacent bands. This physics-based constraint enhances the consistency of representations among adjacent bands, ensuring that subspace representations across all bands maintain well-structured coherence. An efficient algorithm based on the Alternating Direction Method of Multipliers (ADMM) is derived to solve the proposed model. Extensive experiments on three real HSIs demonstrate that ICC-SSC significantly outperforms state-of-the-art methods.

## 1. Introduction

Hyperspectral sensors capture intricate detail of land covers by generating hyperspectral remote sensing image (HSI) cubes with hundreds of contiguous spectral bands, resulting in a wealth of sensor-derived spectral and spatial information [1]. High-resolution HSI information is crucial for effectively distinguishing various ground objects, enabling a wide range of applications such as ocean monitoring [2], land cover classification [3], and object detection [4]. However, the very core capability of these sensors to capture high-dimensional data also presents significant processing challenges. The high dimensionality and strong correlation between adjacent bands inherent in this sensor data often lead to the Hughes phenomenon in classification tasks [5], which can diminish the utility of the sensor’s output. Therefore, developing effective dimensionality reduction techniques is by no means just a data preprocessing step but a crucial requirement to unlock the full potential of hyperspectral sensor technology.

The dimensionality reduction of the HSI primarily involves two distinct approaches: feature extraction [6] and band selection [7,8]. Through feature extraction, the initial high-dimensional spectral bands can be efficiently projected into a low-dimensional space, which facilitates the analysis of HSIs. Instead, band selection methods aim at identifying and extracting a representative subset of bands from the target HSI, thus effectively reducing its dimensionality. The advantage of band selection methods lies in their ability to preserve the original spectral information while ensuring its physical interpretability [8]. Depending on whether training samples are used or not, band selection methods can be classified into three types: supervised [9], unsupervised and semi-supervised [10]. Supervised and semi-supervised methods require labeled samples, whereas unsupervised methods propose a more flexible approach by avoiding this requirement considering the challenge of obtaining labeled data [11].

Unsupervised band selection methods are mainly categorized into ranking-based methods, clustering-based methods, and search-based methods. Ranking-based methods, such as maximum variance-based principal component analysis (MVPCA) [12], fast neighborhood grouping for hyperspectral band selection (FNGBS) [13], and similarity-based ranking strategy [14], typically select the representative band based on specific metrics and their rankings. However, ranking-based methods rarely consider the correlation between bands [15]. Search-based methods commonly address the task of band selection by optimizing a specific objective function, such as adaptive multistrategy particle swarm optimization [16] and the cuckoo search-based method [17]. However, the computational complexity of these methods tends to be high [18]. In terms of clustering-based methods, they perform band selection by grouping similar bands and then selecting a representative band from each group [19], such as enhanced fast density-peak-based clustering (E-FDPC) [20], Ward’s linkage strategy using divergence (WaLuDi) [21], and the adaptive subspace partition strategy (ASPS) [22]. Recently, due to the successful applications of deep learning in processing HSIs [23], deep learning has been used to design effective clustering-based methods for band selection, such as the deep subspace clustering method (DSC) [24] and the sparsity regularized deep subspace clustering method [25]. However, training deep neural network models in these methods requires high-performance devices, and model training is computationally intensive.

Sparse subspace clustering (SSC) [26] has received much attention for its superior performance in hyperspectral band selection. The SSC method first constructs a sparse coefficient matrix assuming that each band of the target HSI can be represented by a linear combination of other bands. Subsequently, a similarity matrix is created from this coefficient matrix. Finally, spectral clustering is performed based on the similarity matrix, and the clustering results obtained are used to select representative bands. Therefore, existing studies mainly enhance the effectiveness of SSC-based methods by improving the quality of the generated sparse coefficient matrices. For example, Sun et al. [27] proposed an SSC-based band selection method that enforces the sparsity of the coefficient matrices and the block diagonal structure. Huang et al. [28] factorized the coefficient matrix of the self-representation model into the desired coefficient matrix and sparse error matrix, and they adaptively adjusted the coefficient matrix to take advantage of the intrinsic structural information in HSIs. Cai et al. [29] incorporated graphical convolution into the self-representation model and provided a closed-form solution. Based on the obtained coefficient matrix, ranking-based and clustering-based strategies are proposed to identify the representative band subset. You et al. [30] presented a global affinity matrix reconstruction (GAMR) method that adaptively reconstructs the global similarity matrix to guide the band selection by iteratively refining pseudo-labels through convex optimization. Although the clustering performance of SSC-based band selection methods has been significantly improved, these methods neglect the high correlation between bands in real HSIs and thus have limited performance. Therefore, it remains challenging to design effective self-representation models by exploiting the inherent high correlation between adjacent bands in real HSIs so as to learn a consistent representation of all bands for effective clustering that contributes to band selection.

To address this inherent challenge in hyperspectral sensor data processing, we propose a novel Interband Consistency-Constrained Structural Subspace Clustering (ICC-SSC) method for hyperspectral band selection. Our approach is grounded in the observation that the spectral signatures of land covers reside in low-dimensional subspaces within the high-dimensional sensor data. We assume that each band within such a material-specific subspace can be represented by a linear combination of a common set of physically informative basis bands. Driven by this sensor-derived physical insight, ICC-SSC innovates in two key aspects: structured sparsity for physical group discovery and physics-aware smoothness constraints, thereby learning more effective band representations. Specifically, instead of the conventional $l_{1}$ norm, we employ the $l_{1,2}$ norm in the self-representation model. This enforcement of structured sparsity ensures that a common set of basis bands collectively reconstructs others within a subspace. This mechanism directly identifies the most representative and physically central bands that anchor the characteristic spectral profiles of distinct materials, leading to a more consistent and interpretable grouping. To explicitly model the high correlation between adjacent bands—a fundamental property of continuous spectral sampling by hyperspectral sensors—we incorporate a total variation (TV) regularization term into the self-representation model. This term penalizes representation discrepancies between neighboring bands, thereby enforcing smoothness along the spectral dimension and ensuring that the learned subspace representations respect the inherent physical continuity of the sensor data. An efficient algorithm based on the Alternating Direction Method of Multipliers (ADMM) algorithm is derived to solve the proposed ICC-SSC model. Finally, based on the cluster partitioning result, we select the most representative and informative bands from each subspace using an information entropy-based criterion to form the representative band subset. The primary contributions of this study are as follows.

- We propose a novel Interband Consistency-Constrained Structural Subspace Clustering (ICC-SSC) method for band selection, replacing the conventional $l_{1}$ norm with the $l_{1,2}$ norm in the self-representation model. This enforces structured sparsity in the coefficient matrix, ensuring that bands within the same subspace share a common set of basis bands for linear representation. The structural constraint enhances intra-subspace consistency and improves clustering discriminability.
- To leverage the inherent high correlation between adjacent bands in hyperspectral imagery (HSI), we integrate total variation (TV) regularization into the self-representation model. This explicitly enforces smoothness and consistency in the representations of neighboring bands.
- We develop an efficient Alternating Direction Method of Multipliers (ADMM)-based optimization strategy to solve the non-convex ICC-SSC model. In addition, the effectiveness of ICC-SSC is demonstrated by an experimental comparison with eight state-of-the-art band selection methods on three real datasets.

This paper is structured as follows: Section 2 briefly describes the sparse subspace clustering model. Next, Section 3 mainly describes the model and optimization solution of the ICC-SSC method proposed in this study. Section 4 outlines the experimental setup, gives a series of experimental results, and discusses the ICC-SSC methodology. Finally, Section 5 summarizes this study.

## 2. Preliminary

In this section, some relevant works on subspace clustering will be briefly introduced, which help to understand the band selection method proposed in this paper.

### 2.1. SSC

SSC aims to group data points that are drawn from a union of multiple low-dimensional subspaces. This is achieved by learning a sparse representation of the data, where each point is reconstructed using a small subset of other points [26]. Specifically, given a high-dimensional data set $Y={\left\{y_{i}\right\}}_{i=1}^{N}$, where $y_{i}\in R^{M}$, the reconstruction of each data point $y_{i}$ is accomplished by effectively combining the other data points in the set Y. Mathematically, the model of SSC is represented as follows:

(1) $$\begin{array}{c} \min_{C}{\left||C|\right|}_{0},\;s.t.\;Y=YC,\;\mathrm{diag}\left(C\right)=0, \end{array}$$

where $||\cdot |{|}_{0}$ indicates the $l_{0}$ norm of matrices; $Y=\left(y_{1},y_{2},\ldots ,y_{i},\ldots ,y_{N}\right)\in R^{M\times N}$ represents the data matrix composed of the data points in the set Y; $C=\left(c_{1},c_{2},\ldots ,c_{i},\ldots ,c_{N}\right)\in R^{N\times N}$ denotes the coefficient matrix consisting of representation vectors of Y, and diag(C)=0, in which $\mathrm{diag}\left(C\right)\in R^{N}$ denotes the vector formed by all diagonal components in C, ensuring that data points in Y are not represented by themselves. It should be noted that each column vector $c_{i}$ in C indicates the relative importance of the other data points in Y when expressing the corresponding data point $y_{i}$ [31].

Mathematically, Equation (1) can be solved by minimizing the objective function ${\left||C|\right|}_{0}$. However, this usually results in an NP-hard problem. Fortunately, with a convex relaxation technique, the $l_{0}$ norm can be replaced by the $l_{1}$ norm, resulting in the sparse self-representation model [26] as follows:

(2) $$\begin{array}{c} \min_{C}{\left||C|\right|}_{1},\;s.t.\;Y=YC,\;\mathrm{diag}\left(C\right)=0. \end{array}$$

### 2.2. $l_{1,2}$ Norm-Based SSC

Recently, the $l_{1,2}$ norm has been successfully applied to SSC problems [32]. Specifically, the row sparsity of C can be achieved by using the $l_{1,2}$ norm on the matrix C so that a common group of base vectors is used to express data points in the same subspace  [33]. Mathematically, the $l_{1,2}$ norm-based SSC model can be expressed by the following:

(3) $$\begin{array}{c} \min_{C}{\left||C|\right|}_{1,2}+\frac{\lambda }{2}{\left||Y-YC|\right|}_{F}^{2},\;s.t.\;\mathrm{diag}\left(C\right)=0, \end{array}$$

where ${\left||C|\right|}_{1,2}=\sum_{i=1}^{N}{\left||C_{i,:}|\right|}_{2}$; λ denotes the regularization parameter.

Then, using the obtained sparse matrix Z, the affinity matrix W can be constructed by the following:

(4) $$\begin{array}{c} W=\left|C\right|+{\left|C\right|}^{T}. \end{array}$$

Next, clustering results are obtained by performing spectral clustering based on W.

## 3. Method

This section describes the model and optimization scheme of the ICC-SSC method proposed in this study. We first introduce a new model of the ICC-SSC method, which aims to obtain clusters that are more favorable for band selection by learning a consistent representation of the bands. Then, the solution method of the ICC-SSC model is analyzed. Furthermore, a strategy for band selection via information entropy is presented based on the clustering results derived from the ICC-SSC method.

### 3.1. Proposed Model

**Figure 1:**
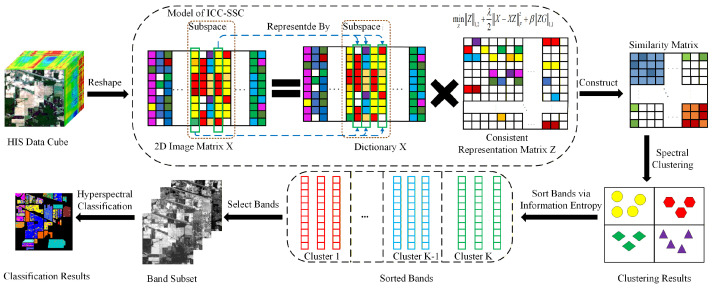
The flowchart of ICC-SSC method, where similar colors represent similar element values.

The overall flowchart of the ICC-SSC method is shown in Figure 1. It can be seen that our proposed ICC-SSC method is achieved through an integrated workflow that begins with the input HSI data cube and progressively identifies the most representative band subset. Central to this process is an autoencoder model regularized by the $l_{1,2}$-norm, which reveals the intrinsic grouping structure of bands by selecting the minimal basis set capable of reconstructing other bands, thereby identifying distinct spectral subspaces. Meanwhile, a TV regularization term is introduced. By promoting smoothness in the representation coefficients, this ensures physical consistency between adjacent bands, enabling the model to directly align with the spectral continuity inherent in the HSI data. This unified model is efficiently optimized using the Alternating Direction Method of Multipliers (ADMM) algorithm, generating a coefficient matrix that encodes subspace membership relationships. Based on the similarity matrix constructed from the obtained coefficient matrix, spectral clustering is then performed to partition the bands into coherent clusters. To robustly select representative bands from each cluster, this method uses information entropy as the final screening criterion. The band with the highest entropy value is chosen as the representative of the cluster, thereby forming a representative subset of bands.

**Figure 2:**
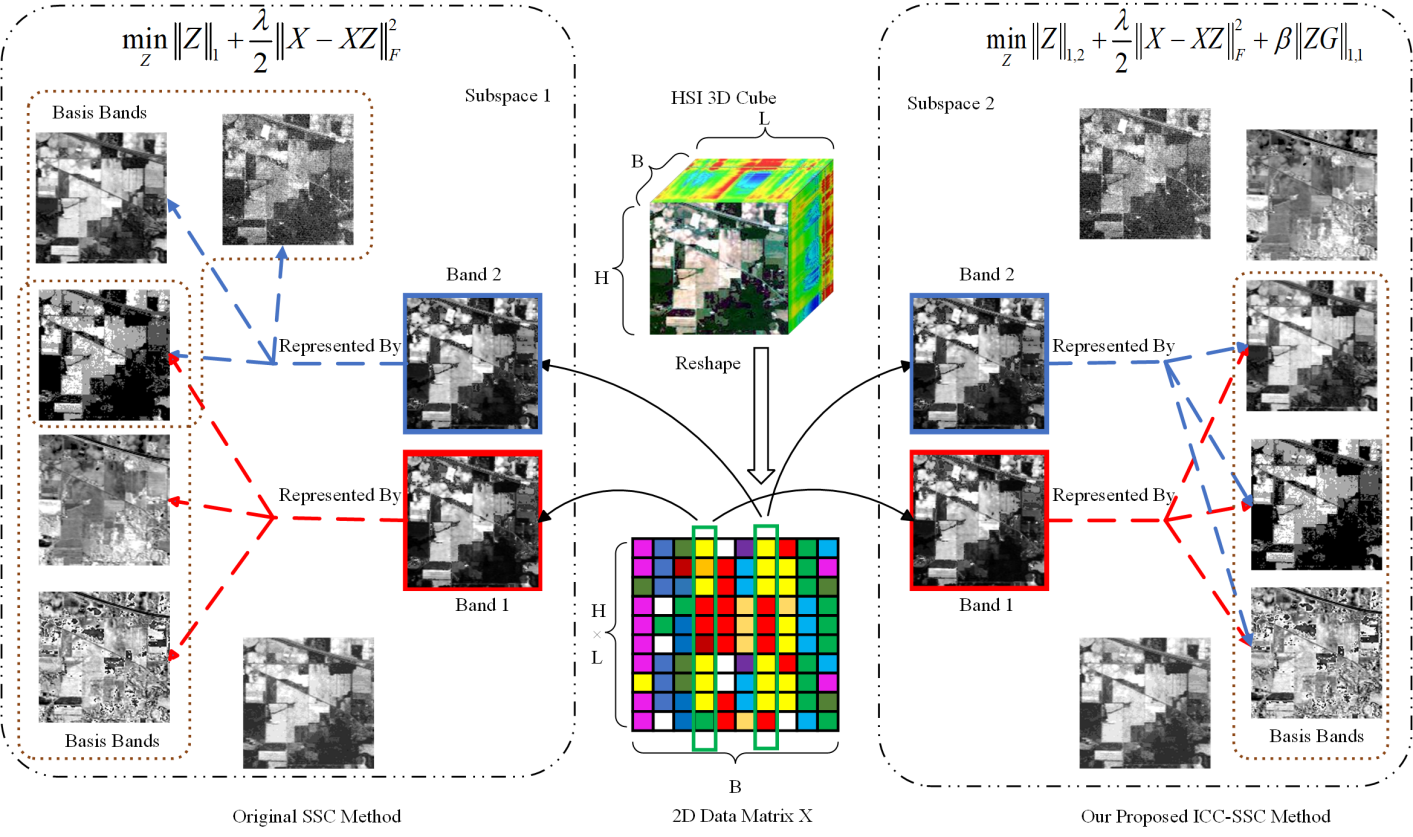
Schematic diagram of ICC-SSC method, where similar colors represent similar element values. Assume that subspace 1 and subspace 2 denote the same subspace of original bands, where band 1 and band 2 represent two neighboring bands in this subspace. In traditional SSC methods, as shown by the dashed arrows, band 1 and band 2 may be represented by different sets of basis bands, which leads to the inconsistent representation of band 1 and band 2. Conversely, the ICC-SSC method can learn a more consistent representation for both bands by employing a common set of basis bands. Specifically, the ICC-SSC approach employs an $l_{1,2}$ norm-based sparse subspace clustering model, which enables the representation of different bands within the same subspace using a common set of basis bands. In addition, the ICC-SSC model ensures consistency in the representation of adjacent bands by integrating total variance regularization.

In addition, Figure 2 illustrates a schematic diagram of the ICC-SSC model, where the different self-representation learning mechanisms between our proposed ICC-SSC method and the traditional SSC method are compared. Prior to sparse self-representation learning, ICC-SSC first reshapes the hyperspectral image cube of size H×W×B into a 2D matrix $X\in R^{M\times B}$, where M=H×W represents the spatial dimensionality, and *H*, *W*, and *B* denote the image height, width, and number of spectral bands, respectively. This is achieved by vectorizing each band image and arranging the resulting vectors in the order of their spectral wavelengths to form the matrix X. In Figure 2, we assume that subspace 1 and subspace 2 denote the same subspace of original bands, where band 1 and band 2 represent two neighboring bands in this subspace. In traditional SSC methods, as shown by the dashed arrows, band 1 and band 2 may be represented by different sets of basis bands, which leads to the inconsistent representation of band 1 and band 2. In contrast, the ICC-SSC method can learn a more consistent representation for both bands by employing a common set of basis bands. Specifically, the ICC-SSC approach employs an $l_{1,2}$ norm-based sparse subspace clustering model, which enables the representation of different bands within the same subspace using a common set of basis bands. Furthermore, the ICC-SSC model incorporates total variation regularization to enforce consistency in the representation of adjacent bands.

Given the obvious interband correlations in HSIs, we introduce a total variation regularization [34] into the $l_{1,2}$ norm-based SSC model [32] to explore the similarity relationship among adjacent bands. Then, the model of ICC-SSC can be expressed as

(5) $$\begin{array}{c} \min_{Z}{\left||Z|\right|}_{1,2}+\frac{\lambda }{2}{\left||X-XZ|\right|}_{F}^{2}+\beta \mathrm{TV}\left(Z\right),\;s.t.\;\mathrm{diag}\left(Z\right)=0, \end{array}$$

where $Z\in R^{B\times B}$ represents the coefficient matrix; $\mathrm{TV}\left(Z\right)=\sum_{j=1}^{B-1}\left|\right|Z_{:,j+1}-Z_{:,j}{\left|\right|}_{1,1}$ denotes the total variation regularization [34], whereas β indicates the regularization parameter. It is worth noting that TV(Z) is used to impose the corresponding constraint that the self-representations of neighboring bands should be similar.

For computational convenience, we construct an auxiliary matrix G with *B* rows and B−1 columns, which is defined as

(6) $$G=\left[\begin{array}{ccccc} 1 & 0 & 0 & \ldots & 0\\ -1 & 1 & 0 & \ldots & 0\\ 0 & -1 & 1 & \ldots & 0\\ 0 & 0 & -1 & \ldots & 0\\ \vdots & \vdots & \vdots & \ddots & \vdots \\ 0 & 0 & 0 & \ldots & 1\\ 0 & 0 & 0 & \ldots & -1 \end{array}\right].$$

Based on the definition of G, the total variation regularization in (5) is rewritten as $\mathrm{TV}\left(Z\right)=||ZG|{|}_{1,1}$, where $||\cdot |{|}_{1,1}$ indicates the $l_{1,1}$ norm of matrices. Given a matrix $C\in R^{M\times N}$, $||C|{|}_{1,1}$ is defined as $||C|{|}_{1,1}=\sum_{n=1}^{N}||C_{:,n}|{|}_{1}$. Accordingly, the model in (5) can be re-expressed as

(7) $$\begin{array}{cc} \; & \min_{Z}{\left||Z|\right|}_{1,2}+\frac{\lambda }{2}{\left||X-XZ|\right|}_{F}^{2}+\beta {\left||ZG|\right|}_{1,1},\;s.t.\;\mathrm{diag}\left(Z\right)=0. \end{array}$$

Considering that the optimization problem shown in (7) is an equality constraint problem, ADMM is an efficient method to solve such problems.

### 3.2. Solution for the Model of ICC-SSC

According to ADMM, to separate the variables, we introduced the auxiliary variables A, $V_{1}$, and $V_{2}$ into the objective function in (7). Thus, the model described in (7) can be reformulated equivalently as

(8) $$\begin{array}{cc} \; & \min_{A,Z,V_{1},V_{2}}{\left||Z|\right|}_{1,2}+\frac{\lambda }{2}{\left||X-XA|\right|}_{F}^{2}+\beta {\left||V_{2}|\right|}_{1,1},\\ \; & s.t.\;A=Z-\mathrm{diag}\left(Z\right),V_{1}=A,V_{2}=AG \end{array}$$

Subsequently, according to the constraints in (8), the corresponding penalty term is added to the objective function to obtain the corresponding Lagrangian function as follows:

(9) $$\begin{array}{cc} \; & L\left(A,Z,V_{1},V_{2},\alpha_{1},\alpha_{2},\alpha_{3}\right)={\left||Z|\right|}_{1,2}+\frac{\lambda }{2}{\left||X-XA|\right|}_{F}^{2}\\ \; & +\beta {\left||V_{2}|\right|}_{1,1}+\frac{\rho }{2}{\left||A-(Z-\mathrm{diag}(Z))|\right|}_{F}^{2}\\ \; & +<\alpha_{1},A-\left(Z-\mathrm{diag}\left(Z\right)\right)>+\frac{\rho }{2}{\left||V_{1}-A|\right|}_{F}^{2}\\ & +<\alpha_{2},V_{1}-A>+\frac{\rho }{2}{\left||V_{2}-V_{1}G|\right|}_{F}^{2}+<\alpha_{3},V_{2}-V_{1}G>, \end{array}$$

where $\alpha_{1}$, $\alpha_{2}$, and $\alpha_{3}$ are three Lagrange multipliers; ρ signifies the penalty coefficient. Based on above Lagrangian function in (9), we iteratively optimize the variables Z, A, $V_{1}$, and $V_{2}$ using ADMM, which is implemented as follows.

Z-update: Based on (9), the variable Z is optimized according to the sub-optimization problem given in (10), where *k* denotes the iteration times.

(10) $$\begin{array}{cc} Z^{\left(k+1\right)}= & \underset{Z}{\arg\min}{\left||Z^{k}|\right|}_{1,2}+\frac{\rho }{2}{\left||A^{k}-\left(Z^{k}-\mathrm{diag}\left(Z^{k}\right)\right)|\right|}_{F}^{2}\\ \; & +<\alpha_{1}^{k},A^{k}-\left(Z^{k}-\mathrm{diag}\left(Z^{k}\right)\right). \end{array}$$

We address the sub-optimization problem from (10) following the Lemma 4.1 in [35], and the resulting update rule about Z is given by the following:

(11) $$\begin{array}{c} Z_{i,:}^{\left(k+1\right)}=\left\{\begin{array}{cc} \frac{||\delta_{i,:}^{k}|{|}_{2}-\zeta }{||\delta_{i,:}^{k}|{|}_{2}}\delta_{i,:}^{k}, & ||\delta_{i,:}^{k}|{|}_{2}\geq \zeta \\ 0, & \mathrm{otherwise}, \end{array}\right. \end{array}$$

where $\zeta =\frac{1}{\rho }$ and $\delta =A^{k}+\frac{\alpha_{1}^{k}}{\rho }$.

A-update: By ignoring the terms in (9) that are unrelated to A, we obtain the sub-optimization problem as shown below:

(12) $$\begin{array}{cc} A^{\left(k+1\right)}= & \underset{A}{\arg\min}\frac{\lambda }{2}{\left||X-XA^{k}|\right|}_{F}^{2}\\ & +<\alpha_{1}^{k},A^{k}-\left(Z^{\left(k+1\right)}-\mathrm{diag}\left(Z^{\left(k+1\right)}\right)\right)>\\ \; & +\frac{\rho }{2}{\left||A^{k}-\left(Z^{\left(k+1\right)}-\mathrm{diag}\left(Z^{\left(k+1\right)}\right)\right)|\right|}_{F}^{2}\\ \; & +\frac{\rho }{2}{\left||V_{1}^{k}-A^{k}|\right|}_{F}^{2}+<\alpha_{2}^{k},V_{1}^{k}-A^{k}>. \end{array}$$

By setting the derivative of the objective function in (12) to zero, we obtain the update rule for A as follows:

(13) $$\begin{array}{c} A^{\left(k+1\right)}={\left(\lambda X^{T}X+2\rho I\right)}^{-1}\left(\lambda X^{T}X+\rho Z^{\left(k+1\right)}+\rho V_{1}^{k}-\alpha_{1}^{k}+\alpha_{2}^{k}\right), \end{array}$$

where $I\in R^{N\times N}$ represents the identity matrix.

$V_{1}$ update: Variable $V_{1}$ is optimized based on the sub-optimization problem shown by (14).

(14) $$\begin{array}{cc} V_{1}^{\left(k+1\right)}=\underset{V_{1}}{\arg\min} & \frac{\rho }{2}{\left||V_{1}^{k}-A^{\left(k+1\right)}|\right|}_{F}^{2}+<\alpha_{2}^{k},V_{1}^{k}-A^{\left(k+1\right)}>\\ \; & +\frac{\rho }{2}{\left||V_{2}^{k}-V_{1}^{k}G|\right|}_{F}^{2}+<\alpha_{3}^{k},V_{2}^{k}-V_{1}^{k}G>. \end{array}$$

By computing the derivative of the objective function in (14) and setting it to zero, we can derive the following update rule for $V_{1}$:

(15) $$\begin{array}{c} V_{1}^{\left(k+1\right)}=\left(A^{\left(k+1\right)}-\frac{\alpha_{2}^{k}}{\rho }+V_{2}^{k}G^{T}+\frac{\alpha_{3}^{k}G^{T}}{\rho }\right){\left(I+GG^{T}\right)}^{-1}. \end{array}$$

$V_{2}$ update: By keeping the variables that are independent of $V_{2}$ constant in (9), we obtain the following sub-optimization problem for $V_{2}$:

(16) $$\begin{array}{cc} V_{2}^{\left(k+1\right)}= & \underset{V_{2}}{\arg\min}\beta {\left||V_{2}^{k}|\right|}_{1,1}+\frac{\rho }{2}{\left||V_{2}^{k}-V_{1}^{\left(k+1\right)}G|\right|}_{F}^{2}\\ \; & +<\alpha_{3}^{k},V_{2}^{k}-V_{1}^{k}G>. \end{array}$$

To solve the sub-optimization problem in (16), we introduce the soft threshold operator [34], and the resulting update rule for $V_{2}$ is expressed as shown below:

(17) $$\begin{array}{c} V_{2}^{\left(k+1\right)}=soft\left(\frac{\beta }{\rho },V_{1}^{\left(k+1\right)}G-\frac{\alpha_{3}^{k}}{\rho }\right), \end{array}$$

where soft(τ,y)≡sign(y)max{|y|−τ,0} represents the soft threshold function.

Based on the obtained $A^{\left(k+1\right)}$, $Z^{\left(k+1\right)}$, $V_{1}^{\left(k+1\right)}$ and $V_{2}^{\left(k+1\right)}$, the update rules for the Lagrange multipliers $\alpha_{1}^{0}$, $\alpha_{2}^{0}$, and $\alpha_{3}^{0}$ are, respectively, given by the following:

(18) $$\begin{array}{cc} \; & \alpha_{1}^{\left(k+1\right)}=\alpha_{1}^{k}+\rho \left(A^{\left(k+1\right)}+Z^{\left(k+1\right)}-\mathrm{diag}\left(Z^{\left(k+1\right)}\right)\right),\\ \; & \alpha_{2}^{\left(k+1\right)}=\alpha_{2}^{k}+\rho \left(V_{1}^{\left(k+1\right)}-A^{\left(k+1\right)}\right),\\ \; & \alpha_{3}^{\left(k+1\right)}=\alpha_{3}^{k}+\rho \left(V_{2}^{\left(k+1\right)}-V_{1}^{\left(k+1\right)}G\right). \end{array}$$

Based on the above update rules, we give the specific algorithmic steps for ICC-SSC as outlined in Algorithm 1. Note that in step 9 of Algorithm 1, ICC-SSC needs to compute the similarity matrix W used for spectral clustering based on the resulting sparse coefficient matrix Z. Inspired by Equation (4) in the literature [36], we calculate W according to the following:

(19) $$\begin{array}{c} W_{i,j}=\frac{Z_{i,j}^{2}+Z_{j,i}^{2}}{Z_{max}}, \end{array}$$

where $Z_{max}$ denotes the largest element in matrix Z, which is used to keep the magnitude of $W_{i,j}$ within a certain range. It should be noted that by squaring the elements of matrix Z in (19), the importance of the bands with higher similarity to band *i* can be increased, while the influence of the bands with lower similarity is reduced, thus strengthening the connection between the bands with higher similarity in the affinity matrix. This helps spectral clustering obtain more accurate segmentation results.
**Algorithm 1:** The Algorithm for ICC-SSC**Input:** the data matrix X, the count of clusters *n*, two regularization parameters λ and β, the tolerance ε, the penalty parameter ρ, and the maximum number of iterations Iter.**Output:** Selected band set Φ.1:Set the number of iterations k=0, initialize $A^{0}$;2:Initialize the Lagrange multipliers $\alpha_{1}^{0}$, $\alpha_{2}^{0}$, and $\alpha_{3}^{0}$;**repeat**3:      Update matrix Z according to (11);4:      Update matrix A by (13);5:      Update matrix $V_{1}$ via (15);6:      Update matrix $V_{2}$ by (17);7:      Update $\alpha_{t}$, where t=1,2,3, according to (18);8:      Set k=k+1;**until** $|Z^{\left(k+1\right)}-Z^{k}|$ < ε or k≥Iter9:Calculate affinity matrix W based on matrix Z according to (19);10:Obtain clustering results via spectral clustering;11:Based on the clustering results, use information entropy to select the target band set Φ;12:**Return:** the selected band set Φ.

### 3.3. Band Selection via Information Entropy

After obtaining the clustering results, selecting representative bands from each cluster is a critical step in selecting bands for HSIs. Due to the fact that noise in hyperspectral images interferes with the affinity matrix and masks the clustering structure, it adversely affects the spectral clustering. Considering that information entropy has good noise adaptation ability [37,38,39], we use information entropy to select bands in the ICC-SSC method.

Specifically, the information entropy for band $X_{:,i}$, where i=1,…,B, is calculated by the following:

(20) $$\begin{array}{c} H\left(X_{:,i}\right)=-\sum_{\omega \in \Omega }p\left(\omega \right)\log p\left(\omega \right), \end{array}$$

where Ω represents the gray space; $H(X_{:,i})$ denotes the information entropy of the band $X_{:,i}$; p(ω) signifies the probability of the gray level ω occurring within the band $X_{:,i}$. Next, we rank all the bands by information entropy and then select a set of most information-rich and top-ranking bands as representative bands. By prioritizing the bands with higher information entropy, the ICC-SSC method ensures that the selected bands are not only differentiated in terms of spectral similarity but also information-rich. In addition, this method also enables ICC-SSC to possess excellent resistance to noise interference.

## 4. Experiments and Discussion

In this section, we focus on the experimental setup and results, and we discuss the ICC-SSC method. We evaluate the ICC-SSC method on three real HSI datasets and compare its performance with eight representative band selection methods.

### 4.1. Datasets

**Figure 3:**
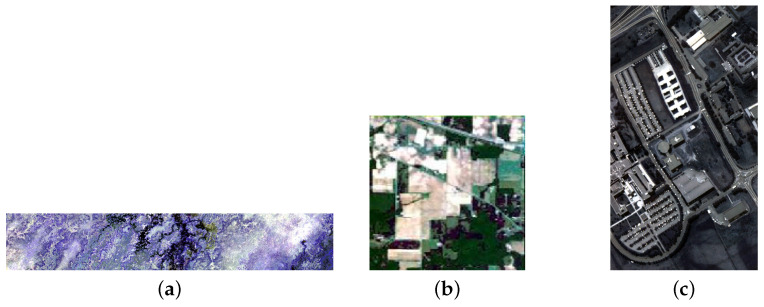
Pseudo-color images of the datasets used in the experiments. (**a**) Botswana dataset. (**b**) Indian Pines dataset. (**c**) Pavia University dataset.

We employ three widely used real HSI datasets in our experiments: the Botswana dataset, the Indian Pine dataset, and the University of Pavia dataset. These datasets are available online (available at https://www.ehu.eus/ccwintco/index.php/Hyperspectral_Remote_Sensing_Scenes, accessed on 1 March 2022). Figure 3 shows the pseudo-color images of the three HSI datasets used in our experiments. Table 1 lists the main information about these three datasets.

The Botswana dataset was collected by the NASA EO-1 satellite during its overflight of Botswana in 2001. This dataset has a spatial resolution of 30 m with a size of 1476 × 256 pixels. After removing the noise band, the remaining 145 bands were employed for our experiments. The study area includes a total of 14 feature classes.

The Indian Pines dataset was obtained by the AVIRIS sensors with the wavelength range of 0.4 to 2.5 μm. This dataset includes 16 categories of land covers and its image has 145 × 145 pixels. In addition, it contains 220 continuous bands with a spatial resolution of about 20 m. After eliminating 20 absorption bands, 200 bands were used in our experiments.

The Pavia University dataset, taken by the ROSIS-03 sensor over the University of Pavia, Italy, is the benchmark HSI dataset for urban remote sensing. This dataset contains 115 spectral bands ranging from 430 nm to 860 nm with a spatial resolution of 1.3 m. This image covers 610 × 340 pixels and contains nine land cover classes such as asphalt, grass and metal sheets.

### 4.2. Experimental Setup

We used two classifiers: Support Vector Machine (SVM) [40] and the K-Nearest Neighbor (KNN) [41] classifier to evaluate the performance of our proposed method for band selection. Note that SVM uses a radial basis function where the penalty parameter value C is set to $1\times 10^{5}$ and the kernel length scale parameter σ is set to 100. The parameter *K* in the KNN classifier is set to 5. The matrix A in the ICC-SSC algorithm is initialized as a matrix of all zeros. In addition, evaluation metrics such as the Overall Accuracy (OA), Average Overall Accuracy (AOA), and Kappa coefficient were used to assess the performance of our method.

To test the performance of the ICC-SSC method, we compared it with eight representative methods, namely, MVPCA [12], WaLuDi [21], E-FDPC [20], DSC [24], ASPS [22], FNGBS [13], GAMR [30], and the tensorial global-local graph self-representation (TGSR) method [42]. Parameter values for all comparison methods were set based on their original studies. During the experiments, the number of bands ranged from 5 to 50 at intervals of 5. The average performance of all methods is reported when tested ten times. Our experiments were carried out on MATLAB 2016b running on a Windows 10 operating system.

### 4.3. Parameter Setting

**Figure 4:**
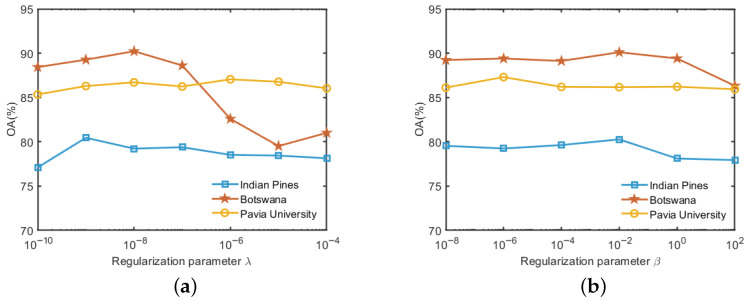
OA variation of two regularization parameters on three datasets. (**a**) Regularization parameter λ. (**b**) Regularization parameter β.

ICC-SSC has two regularization parameters, λ and β. To analyze the effect of the λ and β on the classification performance, we performed experiments using the SVM classifier on two datasets. In our experiments, we analyzed the effect of one parameter by fixing the other. The experimental results are shown in Figure 4. Specifically, Figure 4a shows the effect of the regularization parameter λ when its value changes on the interval $\left[10^{-10},10^{-4}\right]$. From Figure 4a, it can be seen that the OA value is highest when λ is taken as $1\times 10^{-9}$ on the Indian Pines dataset. As the value of λ increases, the OA value decreases slightly. On the Botswana dataset, the OA value is maximum when λ is set to $1\times 10^{-8}$. When $\lambda >10^{-8}$, OA decreases significantly, indicating that Botswana is more sensitive to λ values. In contrast, in the Pavia University dataset, the change in the value of λ has less effect on OA. In addition, Figure 4b shows the results for different values of the regularization parameter β in the range $\left[10^{-8},10^{2}\right]$. It is easy to see from Figure 4b that the maximum value of OA occurs when β is taken as $1\times 10^{-2}$ on the Indian Pines and Botswana data. However, on the Pavia University dataset, OA achieves its maximum value when β is chosen as $1\times 10^{-6}$. When β is set to any other value, the OA values are more similar and slightly worse. This may be due to the fact that a too small value of β will result in the consistency constraint not working, while too large a value of β will lead to a too strong consistency constraint, which will impair the discriminative ability of the feature. Based on this analysis, the regularization parameters λ and β were configured as follows: we set $\beta =1\times 10^{-2}$ and $\lambda =1\times 10^{-9}$ for the Indian Pines dataset, $\beta =1\times 10^{-2}$ and $\lambda =1\times 10^{-8}$ for the Botswana dataset, $\beta =1\times 10^{-6}$ and $\lambda =1\times 10^{-6}$ for the Pavia University dataset.

### 4.4. Experimental Results

#### 4.4.1. Overall Classification Results

**Figure 5:**
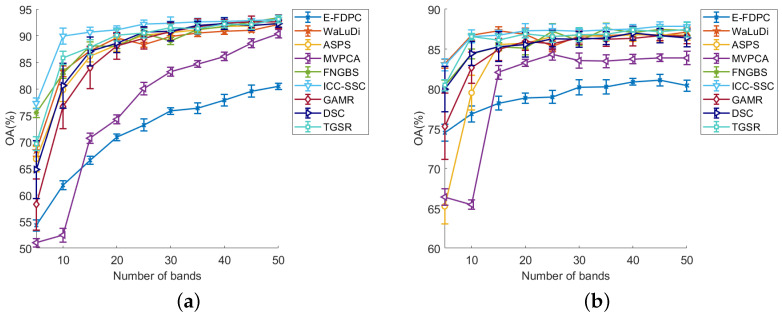
OA values on the Botswana dataset in the conditions of the two classifiers and different numbers of selected bands. (**a**) OA for SVM. (**b**) OA for KNN.

**Figure 6:**
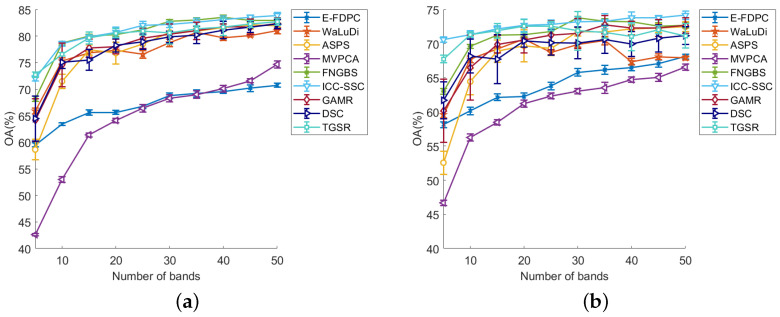
OA values on the Indian Pines dataset in the conditions of two classifiers and different numbers of selected bands. (**a**) OA for SVM. (**b**) OA for KNN.

**Figure 7:**
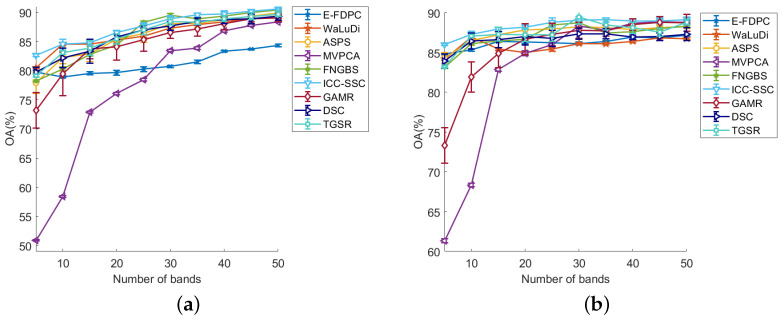
OA values on the Pavia University dataset in the conditions of two classifiers and different numbers of selected bands. (**a**) OA for SVM. (**b**) OA for KNN.

Table 2 shows the average AOA and kappa values obtained by all methods on three datasets, where the values shown in bold red font indicate the best results. From Table 2, we can see that ICC-SSC has the highest classification performance, which is followed by FNBGBS. Specifically, when using the KNN classifier on the Botswana dataset, ICC-SSC slightly outperforms FNBGBS. On the SVM classifier, ICC-SSC achieves significantly better results. When tested on the Indian Tree dataset, ICC-SSC outperforms FNBGBS on both classifiers. Overall, the ICC-SSC outperformed the other eight methods on both data based on AOA and kappa metrics. In addition, Figure 5, Figure 6 and Figure 7 display the curves of OA values across 5 to 50 bands, illustrating the results of all band selection methods tested on different datasets using SVM and KNN classifiers, respectively.

(1) Botswana dataset: Figure 5 presents the OA values for all methods when tested on the Botswana dataset. As seen in Figure 5a, with the SVM classifier, ICC-SSC outperforms other methods for most of the cases. Specifically, ICC-SSC performs best when 5 to 35 bands are selected. When selecting 40 bands, ICC-SSC and GAMR provide comparable best performance. At 45 bands, ICC-SSC performed comparably to GAMR and FNGBS but better than the other methods. With 50 bands selected, ICC-SSC has the second best performance, which was slightly below that of FNGBS. In addition, Figure 5b presents the results of all the methods with the KNN classifier. We can see that ICC-SSC performs best when 20 and 30 bands as well as 40 to 50 bands are selected. When selecting five bands, the performance of ICC-SSC and WaLuDi is comparable but better than those of the other methods. Although ICC-SSC performs similarly to WaLuDi and TGSR in selecting 10 bands, it still outperforms other algorithms. At 25 and 35 bands, ICC-SSC performs similarly to FNGBS and outperforms the other methods. When selecting 15 bands, ICC-SSC ranks second, although its performance falls just below that of WaLuDi. Overall, ICC-SSC performs best on the Botswana dataset. In summary, ICC-SSC offers the best performance.

(2) Indian Pines dataset: Figure 6a,b show the test performance of ICC-SSC and the other eight methods when using SVM and KNN classifiers, respectively, on the Indian Pines dataset. From Figure 6a, we can see that ICC-SSC performs best when 20, 25, 45 and 50 bands are selected. When using 10 and 15 bands, the OA values of ICC-SSC are comparable to those of FNGBS and TGSR while surpassing the OA values of the other methods. At 30, 35 and 40 bands, ICC-SSC ranked second in performance, with FNGBS achieving the top ranking. When selecting five bands, ICC-SSC achieved second place, while TGSR was first. At five bands, the ICC-SSC ranked second in performance, with FNGBS achieving the top ranking. Furthermore, as can be seen from Figure 6b, ICC-SSC performs excellently while using the KNN classifier. Specifically, it performs best with 5, 10, 25 and 40 to 50 bands selected. Although ICC-SSC performs similarly to TGSR in selecting bands 10 and 20, it still outperforms other algorithms. Although ICC-SSC slightly performs worse than FNGBS at 30 bands, it still outperforms all other methods. Overall, ICC-SSC has the best performance.

(3) Pavia University dataset: Figure 7 shows the OA values obtained by all the methods on the Pavia University dataset. From Figure 7a, it can be seen that ICC-SSC shows the best performance when selecting 5, 15, 20, 25, 35 and 40 bands using the SVM classifier. When choosing 10 bands, the best performance is achieved by ICC-SSC, TGSR and WaLuDi. At 45 and 50 bands, the performance of ICC-SSC is comparable to FNGBS and TGSR but better than that of the other methods. With 30 bands, ICC-SSC is slightly lower than FNGBS and TGSR. In addition, Figure 7b presents the results of all the methods with the KNN classifier. As can be seen in the figure, ICC-SSC has the best performance when 5–40 bands are selected. When selecting 40–50 bands, the performance of the ICC-SSC method is comparable to GAMR but superior to the other methods. Overall, ICC-SSC outperforms other comparison methods.

**Figure 8:**
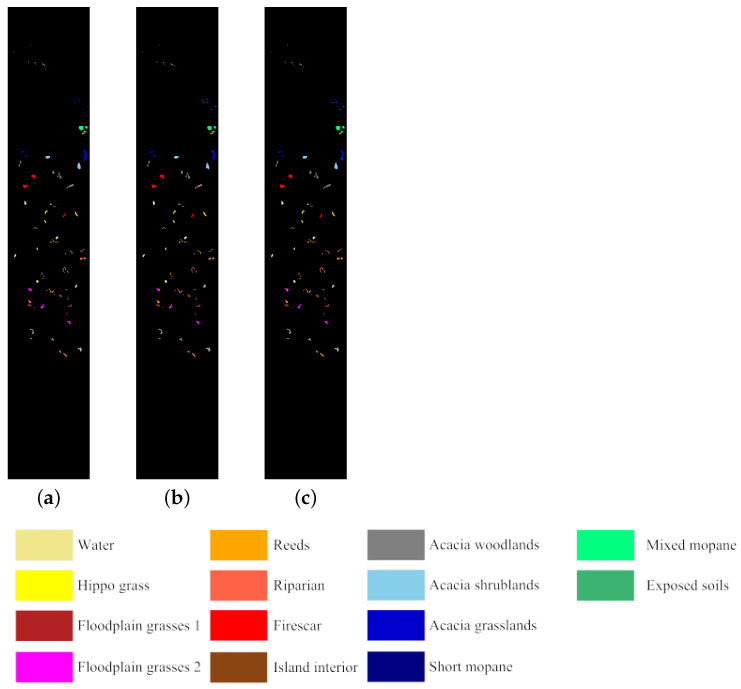
Classification maps from ICC-SSC when 30 bands are selected on the Botswana dataset as well as the ground truth. (**a**) Ground truth. (**b**) Using SVM. (**c**) Using KNN.

**Figure 9:**
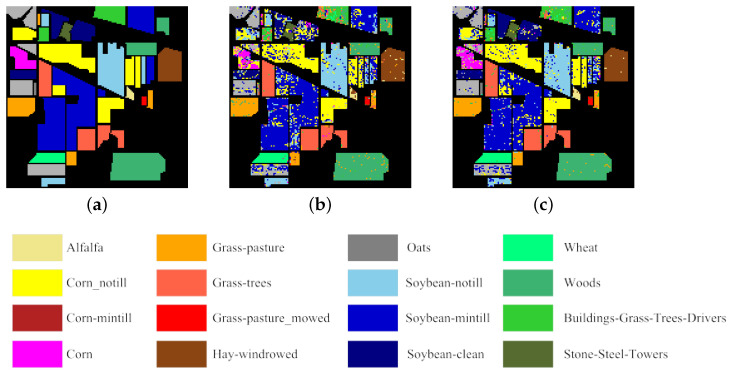
Classification maps from ICC-SSC when 30 bands are selected on the Indian Pines dataset as well as the ground truth. (**a**) Ground truth. (**b**) Using SVM. (**c**) Using KNN.

**Figure 10:**
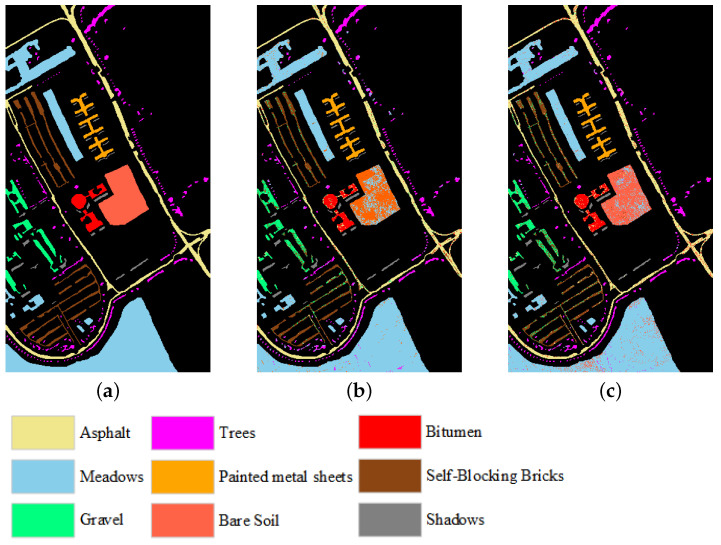
Classification maps from ICC-SSC when 30 bands are selected on the Pavia University dataset as well as the ground truth. (**a**) Ground truth. (**b**) Using SVM. (**c**) Using KNN.

In addition, Figure 8, Figure 9 and Figure 10 compare the differences between the classification maps produced by ICC-SSC using two classifiers and the ground truth when 30 bands are selected, providing an intuitive visualization of the quality of the selected bands. Specifically, Figure 8 shows the classification results obtained using SVM and KNN classifiers on the Botswana dataset compared to the ground truth. Figure 9 presents the results obtained using SVM and KNN classifiers on the Indian Pines dataset compared to the ground truth. Similarly, Figure 10 displays the classification results obtained on the Pavia University dataset using SVM and KNN classifiers compared to the ground truth. Overall, as can be seen from Figure 9 and Figure 10, the results of the ICC-SSC method using SVM and KNN classifiers on three datasets are satisfactory.

#### 4.4.2. Analysis of Per-Class Results

Table 3 lists the detailed performance of ICC-SSC on each class of the Indian Pines dataset, revealing the advantages and specific challenges of agricultural land cover classification. The ICC-SSC method demonstrates excellent performance on several key crop types, achieving outstanding F1-scores for Wheat (0.9415), Grass-trees (0.9535), and Hay-windrowed (0.9571), indicating a reliable discrimination of major agricultural classes. However, the metrics uncover particular difficulties with the Oats class (F1-score: 0.6154), which suffers from low precision (0.5217) despite reasonable recall (0.7500), suggesting issues with false positive detections. Similarly, the Buildings-grass-trees-drives class shows limited performance (F1-score: 0.6690), primarily due to low recall (0.6181), indicating missed detections in this complex mixed-landscape category. The close alignment between macro (0.8182) and weighted (0.8420) F1-scores confirms generally balanced performance across the 16-class agricultural scenario, though the variance in per-class results highlights the challenge of distinguishing spectrally similar crop types at different growth stages.

Table 4 shows the detailed performance of our method on each class of the Pavia University dataset, demonstrating the excellent performance of our method across all categories. As shown in Table 4, our method not only achieved high F1-scores in most categories such as Meadows (0.9428) but also in challenging minority categories such as Shadows (0.9928). High precision values (eight out of nine categories exceeding 0.85) confirm low false positive rates, while consistently high recall values indicate comprehensive detection capabilities. What is particularly impressive is the almost perfect performance on the painted metal plate (F1-score: 1.0000), demonstrating the outstanding ability of our method to distinguish complex urban materials. In addition, the close consistency between macro (0.8860) and weighted (0.8964) F1-scores further validates the balanced performance of classes of different sizes.

### 4.5. Convergence Analysis

**Figure 11:**
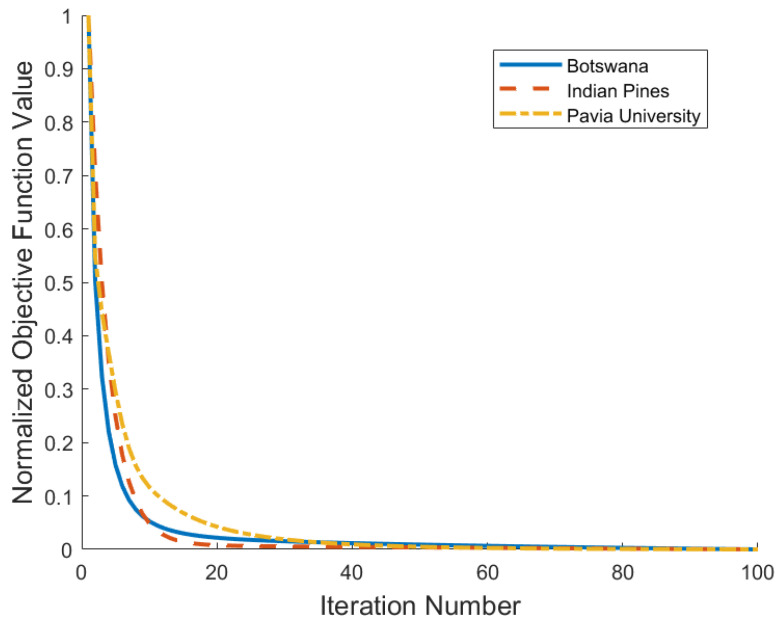
Convergence curve of ICC-SSC.

Figure 11 illustrates the convergence behavior of the proposed ICC-SCC algorithm by plotting the normalized objective function value versus the number of iterations. It is evident that our method exhibits rapid convergence on all three datasets. Specifically, the algorithm converges within 20 iterations for the Indian Pines dataset, and it requires approximately 40 and 45 iterations for the Pavia University and Botswana datasets, respectively. This consistent and efficient convergence across diverse HSI data validates the stability and practicality of the derived ADMM optimization procedure.

### 4.6. Algorithm Complexity Analysis

This section analyzes the computational complexity of the proposed method. According to (7), the optimization problem of ICC-SSC comprises three terms: an $l_{1,2}$-norm regularization term, a Frobenius-norm fitting term, and a TV regularization term, denoted as ${∥ZG∥}_{1,1}$. Under the ADMM framework, the original problem is decomposed into multiple subproblems whose computational complexities are analyzed as follows.

The subproblem for A is a quadratic problem, whose solution is obtained by solving a linear system. Directly solving this system requires $O(B^{3})$ operations, making it the most computationally expensive step per iteration. The subproblem for $V_{1}$ is also a quadratic problem involving the multiplication of B×B matrices with a complexity of $O(B^{3})$. In addition, the updates for Z and $V_{2}$ involve the proximal operators of the $l_{1,2}$-norm and $l_{1,1}$-norm, respectively. These are implemented via group soft-thresholding and element-wise soft-thresholding operations, which each have a complexity of $O(B^{2})$. Moreover, the Lagrange multiplier update is a simple matrix addition with $O(B^{2})$ complexity.

In summary, the per-iteration complexity of the algorithm is dominated by the updates of A and $V_{1}$, each requiring $O(B^{3})$ operations. For a preset number of iterations Iter, the overall computational complexity of the ADMM solver is $O(Iter\cdot B^{3})$. This implies that the computational cost grows cubically with the number of samples *B*, which is a major determinant of the algorithm’s scalability and efficiency.

### 4.7. Discussion

The results shown in Figure 5, Figure 6 and Figure 7 indicate a significant correlation between the number of selected bands and the accuracy of the classifiers. Specifically, the accuracy of the band selection method is positively correlated with the number of selected bands. However, the growth rate of the OA value tends to decrease as the number of selected bands increases. Meanwhile, the experimental results have not demonstrated a linear relationship between the number of selected bands and the classification accuracy of the data, which suggests that there may be a complex interdependence between them, as reported in the literature [43]. This phenomenon is explained by the fact that with increasing number of bands, there is a corresponding increase in the redundancy of information in the dataset.

The ICC-SSC method shows important advantages in terms of the performance and robustness of various classification models. Specifically, our method, especially on SVM and KNN classifiers, shows consistent effectiveness when selecting bands varying between 5 and 50, as shown by Figure 5, Figure 6 and Figure 7. In contrast to other clustering-based methods, ICC-SSC learns a structured sparse representation between bands by self-representation learning while considering the similarity structure between neighboring bands. This enables it to produce effective representations that are more favorable for band selection. This confirms the importance of learning a consistent representation of bands for obtaining effective clustering results [44]. In addition, ICC-SSC benefits from the employed connectivity–improvable affinity matrix, which promotes more effective cluster segmentation. As a result, ICC-SSC achieves excellent band selection performance.

Furthermore, there are some limitations to this study. Specifically, the ICC-SSC method exploits similarity relationships between bands by employing total variation regularization. However, this approach focuses primarily on neighboring bands and thus may not fully capture the overall similarity relationship across all bands. Secondly, the performance of the ICC-SSC model is significantly affected by its three key parameters, namely the regularization parameters λ and β, as well as the penalty parameter ρ, which significantly affect the validity and applicability of our approach, and whose effective values may be data-dependent. Therefore, investigating adaptive tuning strategies for these parameters and developing methods that can more effectively exploit the global similarity properties of HSIs between bands are important directions for further research.

## 5. Conclusions

In this paper, we proposed a novel Interband Consistency-Constrained Structural Subspace Clustering (ICC-SSC) approach for hyperspectral band selection. This method is specifically designed to address the data redundancy challenge inherent in hyperspectral sensor data by leveraging two key physical properties: the intrinsic self-representation characteristic of the data and the high correlation among neighboring bands. The core of our contribution lies in its sensor-data-centric design. By employing the $l_{1,2}$ norm, our model learns a structured sparse representation that effectively identifies a common set of physically informative basis bands. This ensures that the resulting band groups are not only compact but also spectrally representative and interpretable. Furthermore, the incorporation of TV regularization explicitly enforces smoothness along the spectral dimension, which is a direct reflection of the continuous sampling nature of hyperspectral sensors. This physics-aware constraint significantly enhances the consistency between adjacent bands, leading to a more physically coherent and stable band grouping structure.

The efficacy of our method was validated through extensive experiments. The results demonstrate that ICC-SSC significantly outperforms state-of-the-art methods, proving its capability in selecting a highly discriminative and non-redundant band subset. This work underscores the critical role of advanced, physics-driven algorithms in maximizing the practical utility of hyperspectral sensors. By transforming high-dimensional, raw sensor output into a compact yet physically meaningful set of bands, our method facilitates more efficient and accurate downstream applications, thereby enhancing the value proposition of hyperspectral sensor technology itself.

## Figures and Tables

**Table 1:** Specific information of the HSI datasets used.

Dataset	Pixels	Spatial Resolutions	Classes	Bands
Botswana	1476 × 256 pixels	30 m/pixel	14	145
Indian Pines	145 × 145 pixels	20 m/pixel	16	200
Pavia University	610 × 340 pixels	1.3 m/pixel	9	103

**Table 2:** Performance comparison of all methods on three datasets. The best results are shown in red bold font.

Dataset	Classifier	ICC-SSC	MVPCA (1999) [12]	WaLuDi (2007) [21]	E-FDPC (2016) [20]	DSC (2019) [24]	ASPS (2019) [22]	FNGBS (2021) [13]	GAMR (2023) [30]	TGSR (2024) [42]
Botswana	AOA (SVM)	**90.482 ± 0.314**	76.147 ± 0.444	87.105 ± 0.264	71.678 ± 0.444	87.107 ± 0.746	86.885 ± 0.389	88.271 ± 0.131	87.418 ± 0.811	88.419 ± 0.289
	Kappa (SVM)	**0.8823 ± 0.0034**	0.7416 ± 0.0048	0.8603 ± 0.0029	0.6934 ± 0.0048	0.8604 ± 0.0081	0.8579 ± 0.0042	0.8730 ± 0.0014	0.8637 ± 0.0088	0.8745 ± 0.0031
	AOA (KNN)	**86.886 ± 0.204**	80.010 ± 0.136	86.331 ± 0.175	79.021 ± 0.319	85.417 ± 0.354	83.620 ± 0.245	86.568 ± 0.314	85.885 ± 0.748	86.2287 ± 0.192
	Kappa (KNN)	**0.8575 ± 0.0022**	0.7834 ± 0.0015	0.8519 ± 0.0019	0.7729 ± 0.0034	0.8420 ± 0.0038	0.8226 ± 0.0025	0.8461 ± 0.0034	0.8341 ± 0.0081	0.851 ± 0.0021
Indian Pines	AOA (SVM)	**80.865 ± 0.350**	64.263 ± 0.217	77.433 ± 0.217	64.126 ± 0.098	77.740 ± 0.687	77.509 ± 0.477	80.044 ± 0.189	76.974 ± 0.545	79.917 ± 0.544
	Kappa (SVM)	**0.7818 ± 0.0038**	0.5853 ± 0.0021	0.7397 ± 0.0020	0.6199 ± 0.0011	0.7455 ± 0.0079	0.7372 ± 0.0049	0.7758 ± 0.0022	0.7497 ± 0.0066	0.7709 ± 0.0062
	AOA (KNN)	**72.786 ± 0.313**	60.795 ± 0.180	67.953 ± 0.124	64.017 ± 0.204	69.081 ± 0.771	68.531 ± 0.497	71.248 ± 0.151	69.980 ± 0.936	71.384 ± 0.779
	Kappa (KNN)	**0.6884 ± 0.0035**	0.5505 ± 0.0019	0.6327 ± 0.0014	0.5863 ± 0.0014	0.6457 ± 0.0090	0.6395 ± 0.0056	0.6705 ± 0.0017	0.6558 ± 0.0108	0.6720 ± 0.0091
Pavia University	AOA (SVM)	**87.527 ± 0.168**	76.708 ± 0.076	86.246 ± 0.091	81.176 ± 0.105	86.117 ± 0.388	85.899 ± 0.2153	86.223 ± 0.104	84.544 ± 0.637	86.334 ± 0.305
	Kappa (SVM)	**0.8340 ± 0.0023**	0.6683 ± 0.0011	0.8168 ± 0.0012	0.7495 ± 0.0014	0.8150 ± 0.0051	0.8120 ± 0.0028	0.8164 ± 0.0014	0.7931 ± 0.0062	0.8180 ± 0.0040
	AOA (KNN)	**88.353 ± 0.087**	82.592 ± 0.067	85.931 ± 0.054	86.268 ± 0.066	86.680 ± 0.276	87.456 ± 0.212	87.099 ± 82.554	87.506 ± 0.649	87.448 ± 0.0028
	Kappa (KNN)	**0.8426 ± 0.00133**	0.7622 ± 0.0008	0.8095 ± 0.0007	0.8141 ± 0.0009	0.8199 ± 0.0032	0.8305 ± 0.0029	0.8255 ± 0.0006	0.8315 ± 0.0089	0.8274 ± 0.0039

**Table 3:** Detailed per-class performance on the Indian Pines dataset.

Class	Precision	F1-Score	Recall
Alfalfa	0.7234	0.8095	0.9189
Corn-notill	0.7563	0.7993	0.8476
Corn-mintill	0.7636	0.7513	0.7395
Corn	0.7348	0.7170	0.7000
Grass-pasture	0.8646	0.9021	0.9430
Grass-trees	0.9258	0.9535	0.9829
Grass-pasture-mowed	0.7143	0.6977	0.6818
Hay-windrowed	0.9808	0.9571	0.9346
Oats	0.5217	0.6154	0.7500
Soybean-notill	0.8267	0.7987	0.7725
Soybean-mintill	0.8302	0.8296	0.8289
Soybean-clean	0.8670	0.8308	0.7975
Wheat	0.9045	0.9415	0.9817
Woods	0.9487	0.9305	0.9130
Buildings-grass-trees-drives	0.7290	0.6690	0.6181
Stone-steel-towers	1.0000	0.8955	0.8108
Macro average	0.8187	0.8182	0.8263
Weighed average	0.8401	0.8420	0.8406

**Table 4:** Detailed per-class performance on the Pavia University dataset.

Class	Precision	F1-Score	Recall
Asphalt	0.9188	0.9075	0.9131
Meadows	0.9506	0.9352	0.9428
Gravel	0.7195	0.7402	0.7297
Trees	0.9323	0.9485	0.9403
Painted metal sheets	0.9963	1.0000	0.9981
Bare soil	0.7773	0.8128	0.7947
Bitumen	0.8534	0.8423	0.8478
Self-blocking bricks	0.7811	0.7979	0.7894
Shadows	0.9960	0.9895	0.9928
Macro average	0.8832	0.8860	0.8806
Weighed average	0.8968	0.8964	0.8975

## Data Availability

Data are contained within the article.

## Abbreviations

The following abbreviations are used in this manuscript:

HSI	Hyperspectral remote sensing image
SSC	Sparse subspace clustering
ICC-SSC	Interband consistency-constrained structural subspace clustering
ADMM	Alternating direction multiplier method
WaLuDi	Ward’s linkage strategy using divergence
MVPCA	Maximum-variance principal component analysis
ASPS	Adaptive subspace partition strategy
E-FDPC	Enhanced fast density-peak-based clustering
FNGBS	Fast neighborhood grouping method for hyperspectral band selection
GAMR	Global affinity matrix reconstruction
DSC	Deep subspace clustering method
TGSR	Tensorial global–local graph self-representation
SVM	Support vector machine
KNN	K-nearest neighbor
OA	Overall accuracy
AOA	Average overall accuracy
